# Clinical outcomes of bronchodilator use in bronchiolitis: an evidence-based perspective

**DOI:** 10.1097/MS9.0000000000005210

**Published:** 2026-06-02

**Authors:** Farhan Ahmed, Abdul Ghafoor Qureshi, Syed Shahmeer Ahmed, Muhammad Tauseef Haider Malik, Abedin Samadi

**Affiliations:** a Department of Medicine, Karachi Medical and Dental College, Karachi, Pakistan; b Department of Medicine, Dow University of Health Sciences, Karachi, Pakistan; c Department of Medicine, Kabul University of Medical Sciences Abu Ali Sina, Kabul, Afghanistan

**Keywords:** bronchiolitis, bronchodilators, respiratory syncytial virus, salbutamol, supportive care

## Abstract

Bronchiolitis is a leading cause of lower respiratory tract infection in infants, most commonly associated with respiratory syncytial virus, and contributes significantly to global pediatric morbidity. Despite its high prevalence, management remains largely supportive, as no pharmacologic therapy has consistently demonstrated a reduction in disease duration or hospitalization outcomes. This review evaluates the clinical efficacy of bronchodilators, particularly salbutamol, in the treatment of bronchiolitis based on current evidence. While some randomized controlled trials report short-term improvements in clinical scores following bronchodilator administration, pooled data from meta-analyses indicate no significant benefit in key clinical outcomes, including oxygen saturation, hospitalization rates, length of hospital stay, or time to resolution of illness. Furthermore, bronchodilator use has been associated with adverse physiological effects, such as increased heart rate and respiratory rate. The underlying pathophysiology of bronchiolitis, characterized primarily by airway obstruction due to inflammation, edema, and mucus plugging rather than bronchospasm, explains the limited efficacy of bronchodilators. Current international guidelines strongly discourage their routine use and emphasize supportive care as the cornerstone of management. Bridging the gap between evidence and clinical practice is essential to avoid unnecessary interventions and optimize patient outcomes.

## Introduction

Bronchiolitis is a lower respiratory tract condition caused by a viral infection that primarily affects newborns in their first 2 years of life^[^1^]^. Globally, bronchiolitis is a major source of morbidity and mortality, primarily due to the respiratory syncytial virus (RSV). Infants fewer than 2 months of age have a significantly higher hospitalization rate of 17.9 per 1000 children, compared to the average RSV hospitalization rate of 5.2 per 1000 children under 24 months of age in the United States. After 2–4 days of low-grade fever, rhinorrhea, and nasal congestion, young children with bronchiolitis usually show more signs of respiratory tract illness (cough, wheezing, tachypnea, and increased breathing effort)^[^2^]^.

## Pathophysiology and current management

Acute inflammation, mucosal and submucosal edema, necrosis of the tiny airway epithelial cells, increased mucus output, and poor mucus clearance are the main findings in the pathophysiology of bronchiolitis. Patients eventually experience reduced gas exchange as a result of these alterations obstructing their airways. It is challenging to choose the appropriate course of action for infants with bronchiolitis because there are currently no treatments that can lessen the illness’s duration or the length of hospital stay, despite the fact that bronchiolitis can pose a health risk to young children. The most common treatment for young children with bronchiolitis is supportive care, which includes supplemental oxygen as well as proper nourishment and hydration. Corticosteroids and antimicrobial medications are not recommended^[^1^]^.

## Evidence supporting bronchodilator use

A randomized controlled trial found that nebulized salbutamol treatment improves the clinical scores of young children with acute bronchiolitis, including infants under 1 year old and those infected with RSV. The difference between patients in the salbutamol and placebo arms was most significant 30 minutes after the initial medication^[^3^]^.

## Evidence against bronchodilator use

Many people believe that bronchodilator therapy is unlikely to be effective in infants with RSV bronchiolitis^[^4^]^ because evidence from postmortem examinations indicates that the mechanical effect of cellular debris in the bronchial lumen is the main cause of the airway obstruction^[^5^]^ with bronchial smooth muscle spasm seeming to play an insignificant role^[^6^]^.

## Effects of bronchodilators on clinical outcomes

Following are the effects of bronchodilators:

1. **Effects on respiratory rate**

A pooling of data from five studies found that infants treated with salbutamol experienced an overall statistically significant increase in respiratory rate [weighted mean difference (WMD) 2.26 (95% confidence interval [CI] 0.36–4.16)]^[^1^]^.

**2. Effects on heart rate**

When compared to a placebo, the outcomes indicated that infants receiving salbutamol had a significantly higher heart rate [WMD 12.15 (95% CI 9.24–15.07)] Several studies have shown that salbutamol can raise heart rate and oxygen saturation levels in infants with bronchiolitis^[^1^]^.

**3. Effects on oxygen saturation**

The oxygen saturation level in infants was not statistically improved by the administration of salbutamol [WMD 0.20 (95% CI: −0.35 to 0.75)]^[^1^]^.When compared to placebo, bronchodilator users’ oxygen saturation, as determined by pulse oximetry, did not significantly improve, as reflected by the WMD −0.43, 95% CI −0.92 to 0.06^[^2^]^.

**4. Effects on clinical severity score**

A meta-analysis of six of these studies found no indication of a statistically significant improvement in clinical severity scores in infants treated with salbutamol compared to those without treatment [WMD 0.11 (95% CI −0.26 to 0.03)]^[^1^]^.

**5. Admission to hospital**

The rate of hospitalization was not significantly reduced among bronchodilator patients compared to placebo recipients in outpatient studies (11.9% vs 15.9%; OR 0.75, 95% CI 0.46–1.21)^[^2^]^.

## Effects on length of hospitalization

The results of the analysis revealed that neither nebulized nor oral salbutamol medication demonstrated effects on infants with bronchiolitis in terms of lowering the period of hospitalization compared to the control group [WMD 0.12 (95% CI −0.32 to 0.56)]^[^1^]^. The length of stay did not differ between the bronchodilator and placebo groups [MD 0.06 days, (95% CI −0.27 to 0.39)]^[^2^]^. Although a prior systematic review suggested that using nebulized epinephrine for bronchiolitis may reduce hospital admissions, there is little to no evidence that it shortens hospital stays^[^7^]^. There is currently not enough data to warrant the regular use of nebulized hypertonic saline or epinephrine. Although they may marginally improve clinical symptom scores in the near term, beta-2 agonists, such as salbutamol (albuterol), are not recommended because they do not shorten hospital stays^[^1^]^.

## Time to resolution

Long-term home-based studies by Patel^[^8^]^ and Gupta^[^9^]^ found no significant difference in time to resolution of illness between the bronchodilator and placebo groups [MD 0.29, 95% CI −0.43 to 1.00, *n* = 269). Therefore, in infants receiving at-home treatment, oral bronchodilators do not shorten the duration of illness resolution. Two meta-analyses of albuterol use in bronchiolitis found that it has no effect on clinically significant outcomes such as oxygen consumption, hospitalization, or length of stay when compared to placebo^[^10,11^]^. A meta-analysis of epinephrine efficacy found that evidence supports only short-term improvements in clinical scoring and physiologic markers, with no compelling influence on outcomes^[^12^]^.

## Guideline recommendations

According to the Cincinnati guidelines, no bronchodilators, steroids, antivirals, or antibacterial medicines should be used routinely^[^13^]^.

The Canadian Paediatric Society’s updated guidelines do not recommend salbutamol therapy.

## Summary and conclusion

Bronchiolitis remains a primary cause of infant morbidity worldwide, although its treatment continues to deviate from evidence-based guidelines. Strong data from randomized trials and meta-analyses consistently show that bronchodilators, like salbutamol, do not improve significant clinical outcomes, such as oxygen saturation, hospitalization rates, length of stay, or time to resolution, despite brief improvements in clinical scores observed in some trials. Furthermore, their use is associated with negative physiological effects, most notably higher heart rate and respiration rate.

Current international guidelines, such as those from Cincinnati Children’s Hospital and the Canadian Paediatric Society, strongly discourage the routine use of bronchodilators in bronchiolitis, emphasizing supportive care as the cornerstone of management. However, the overuse of bronchodilators continues in clinical practice, indicating a substantial discrepancy between recommendations and reality.

To minimize unnecessary interventions and potential injury in this vulnerable population, bridging the gap requires targeted physician education, adherence to standard guidelines, and a shift toward evidence-based, supportive care.

To ensure methodological transparency and ethical compliance in manuscript preparation, we adhered to the recently developed TITAN (Transparency in the Reporting of Artificial Intelligence)^[^13^]^ guidelines, which outline standards for responsible artificial intelligence use in biomedical writing and publishing^[^14^]^.

## Data Availability

Not applicable.
